# Supplementary material for: Rbpj expression in regulatory T cells is critical for restraining TH2 responses
Source: Nat Commun. 2019 Apr 8;10:1621. doi: 10.1038/s41467-019-09276-w (PMC6453958; doi:10.1038/s41467-019-09276-w)
Supplement: Supplementary file 1 — Supplementary Information [file 41467_2019_9276_MOESM1_ESM.pdf]

Supplementary Information

RBPJ expression in regulatory T cells is critical for restraining T_H2 responses

Michael Delacher, Christian Schmidl, Yonatan Herzig, Minka Breloer, Wiebke Hartmann, Fabian Brunk, Danny Kägebein, Ulrike Träger, Ann-Cathrin Hofer, Sebastian Bittner, Dieter Weichenhan, Charles D. Imbusch, Agnes Hotz-Wagenblatt, Thomas Hielscher, Achim Breiling, Giuseppina Federico, Hermann-Josef Gröne, Roland M. Schmid, Michael Rehli, Jakub Abramson and Markus Feuerer

Supplementary Figure 1

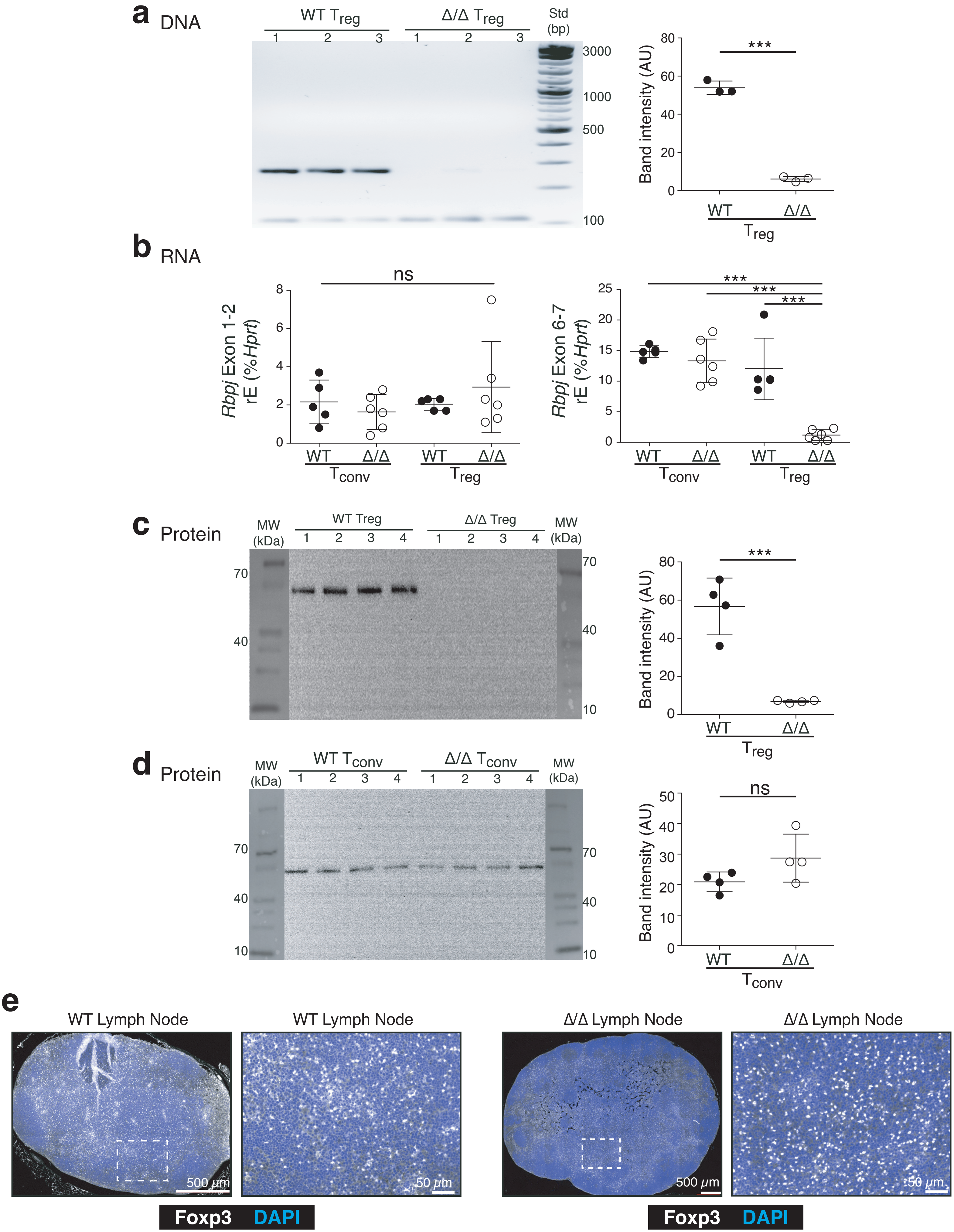

Supplementary Figure 1. Confirmation of *Rbpj* deletion specificity in T_{reg} cells and immunohistochemistry of lymph nodes. This supplementary figure is an extension of **Figure 1**. (**a**) Isolation of genomic DNA from FACS-isolated T_{reg} ($CD3^+CD4^+CD8^-CD25^+Foxp3\text{-}YFP^+$) and T_{conv} ($CD3^+CD4^+CD8^-CD25^-Foxp3\text{-}YFP^-$) cells from WT or Δ/Δ animals followed by PCR amplification of *Rbpj* exon 6-7 region. PCR product in WT cells is expected at around 250 bp. Quantification of band staining intensity was performed with ImageJ software and plotted to the right (n=3, unpaired t test). (**b**) Real-time PCR measurement of *Rbpj* exon 1/2 and exon 6/7 with reversely-transcribed RNA isolated from T_{reg} and T_{conv} cells sorted from WT or Δ/Δ animals. Statistical quantification with one-way ANOVA and Bonferroni post-test (n=5-6). (**c, d**) Western Blot with FACS-isolated T_{reg} (**c**) and T_{conv} (**d**) cells isolated from WT or Δ/Δ animals. We used an antibody against Rbpj followed by chromogenic detection. Quantification of band staining intensity with ImageJ software, plotted to the right (n=4, unpaired t test). (**e**) Immunohistochemistry of whole lymph nodes from WT (left) vs. affected Δ/Δ animals (right). Left-handed images show the whole lymph node with a rectangular section, while the right-hand images are a magnification of this rectangular selection. Foxp3 staining in white, DAPI nuclear staining in blue. Color intensities of images have been adjusted individually. Source data are provided as a Source Data file.

Supplementary Figure 2

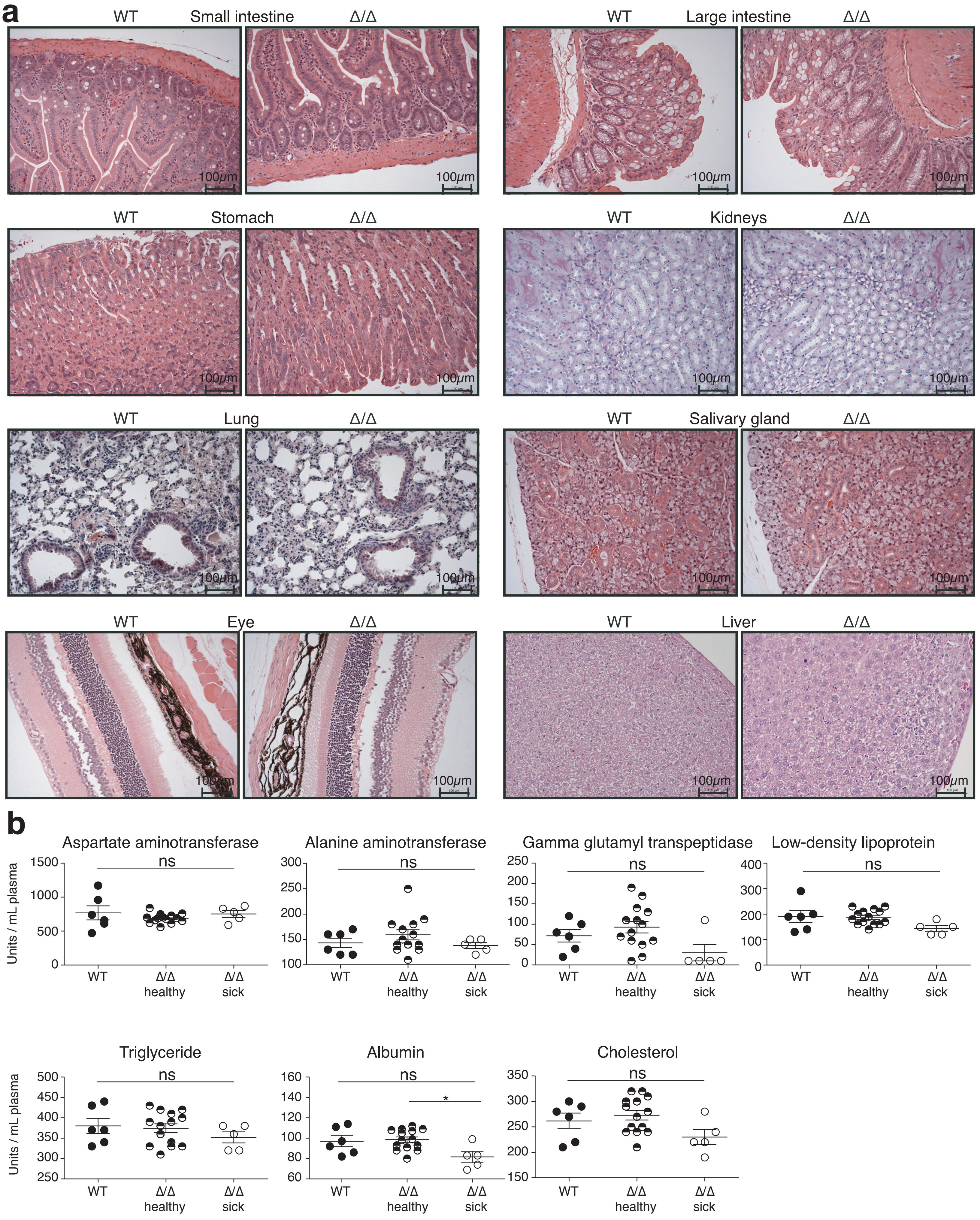

Supplementary Figure 2. Organ pathology and peripheral blood serum parameters. This supplementary figure is an extension of **Figure 1.** (a) H&E staining of small intestine, large intestine, stomach, lung, salivary gland, eye and liver in WT and Δ/Δ animals; Periodic Acid-Schiff staining of kidneys. ≥ 5 animals of each genotype were observed. Representative images are shown. (**b**) Peripheral blood serum was collected from six healthy WT animals, fourteen healthy Δ/Δ animals, and five affected Δ/Δ animals. Blood parameters were statistically evaluated by one-way ANOVA with Bonferroni post-test (*=p<0.05; ns=p>0.05). We measured AST (aspartate aminotransferase), ALT (Alanine aminotransferase), GGT (Gamma glutamyl transpeptidase), LDL (Low-density lipoprotein), triglyceride, albumin and cholesterol levels. Source data are provided as a Source Data file.

Supplementary Figure 3

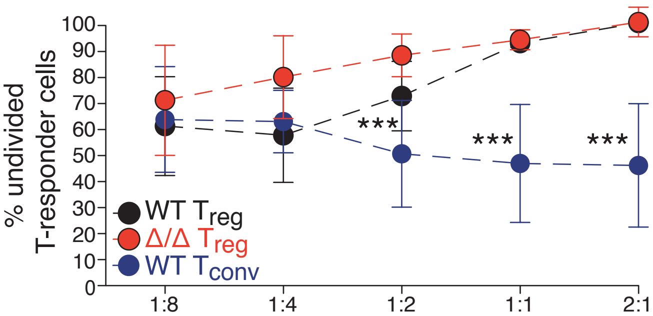

Supplementary Figure 3. *In-vitro* Treg suppression assay with T_{reg} cells from affected Δ/Δ animals vs WT animals. *In-vitro* T_{reg} suppression assay with T_{reg} or T_{conv} cells isolated from WT (black) or affected Δ/Δ (red) animals. X-axis T_{reg} or T_{conv} to T-responder cell ratio. T-responder cells isolated from congenic donor animals, CFSE-labeled and stimulated with antigen-presenting cells plus anti-CD3 antibody. Y-axis percentage of undivided T-responder cells. Statistical analysis two-way ANOVA and Bonferroni post-test (n=6). Source data are provided as a Source Data file.

Supplementary Figure 4

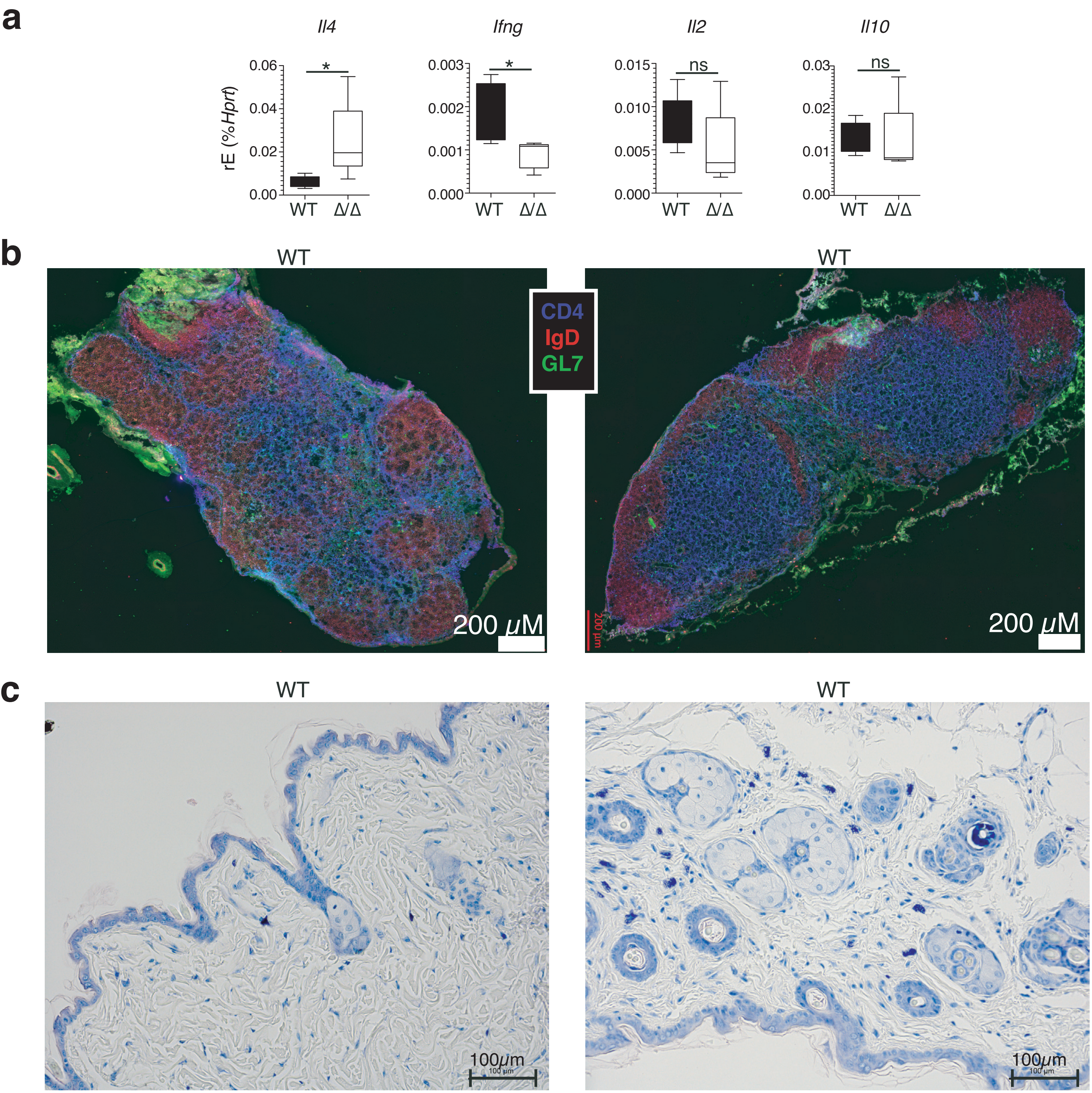

Supplementary Figure 4. Lymph node gene expression, germinal center formation, and Giemsa staining of skin. This supplementary figure is an extension of **Figure 2**. **(a)** qPCR expression of target genes in lymph nodes. Plotted are gene expression values for *Il4*, *Ifng*, *Il2*, *Il10* in WT (black) and affected Δ/Δ (white) animal-derived lymph nodes (n=5, unpaired t test). (**b**) This is an extension of **Figure 2d**. Additional immunohistochemistry of LN from WT mice. CD4 staining in blue, IgD staining in red, and GL7 staining in green. All images have been recorded with same settings and color intensity adjustments have been performed for all images similarly. (**c**) This is an extension of **Figure 2e**. Giemsa staining of skin tissue from two WT animals. Mast cells are stained in blue. In IHC and Giemsa stainings, original magnification scale bars magnified for better visibility. Source data are provided as a Source Data file.

Supplementary Figure 5

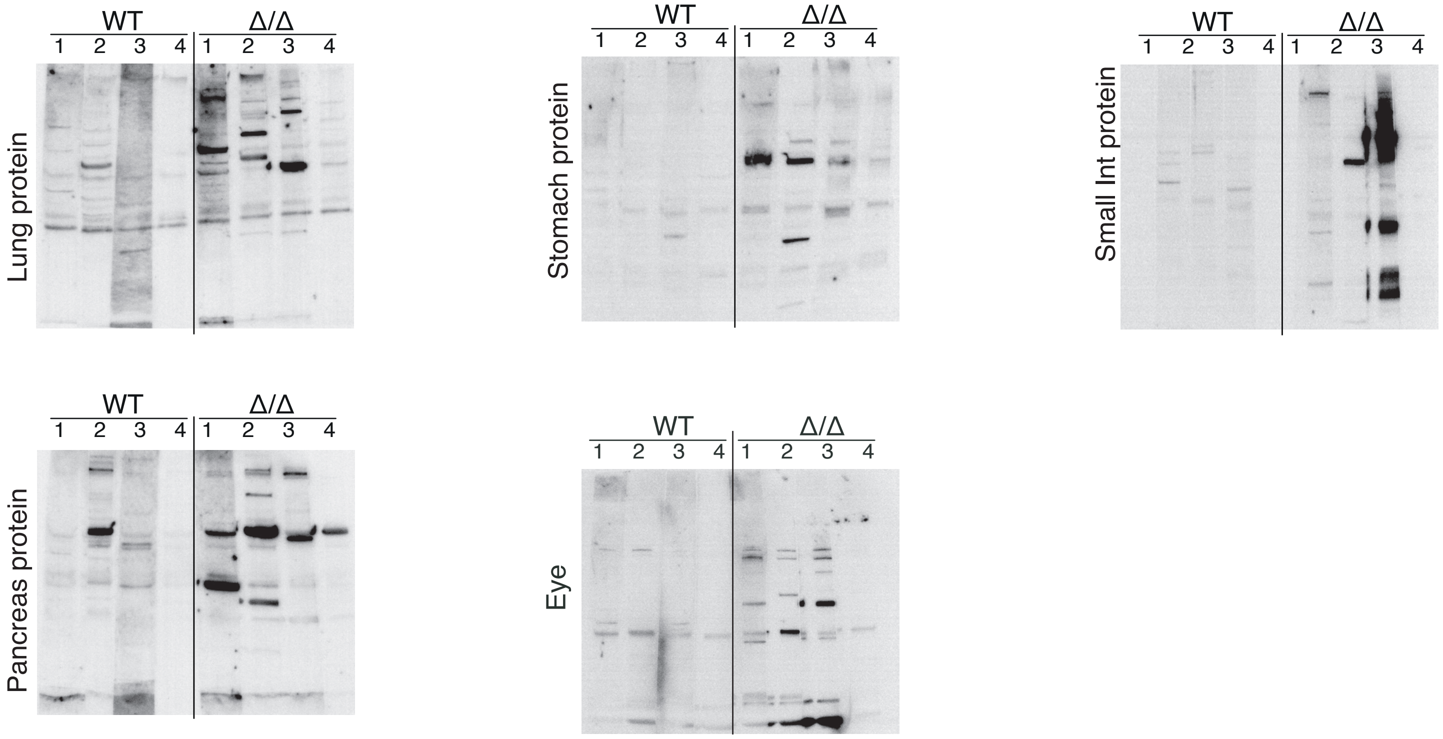

Supplementary Figure 5. Autoantibody detection with blood serum from affected Δ/Δ animals vs WT animals. This is an extension of **Figure 2**. Western Blot with serum from WT and affected Δ/Δ animals. Protein (lung, stomach, small intestine, pancreas or eye) from Rag2-deficient animals has been separated by SDS-PAGE and blotted, followed by incubation with blood serum and chromogenic detection of antibody binding (n=4).

Supplementary Figure 6

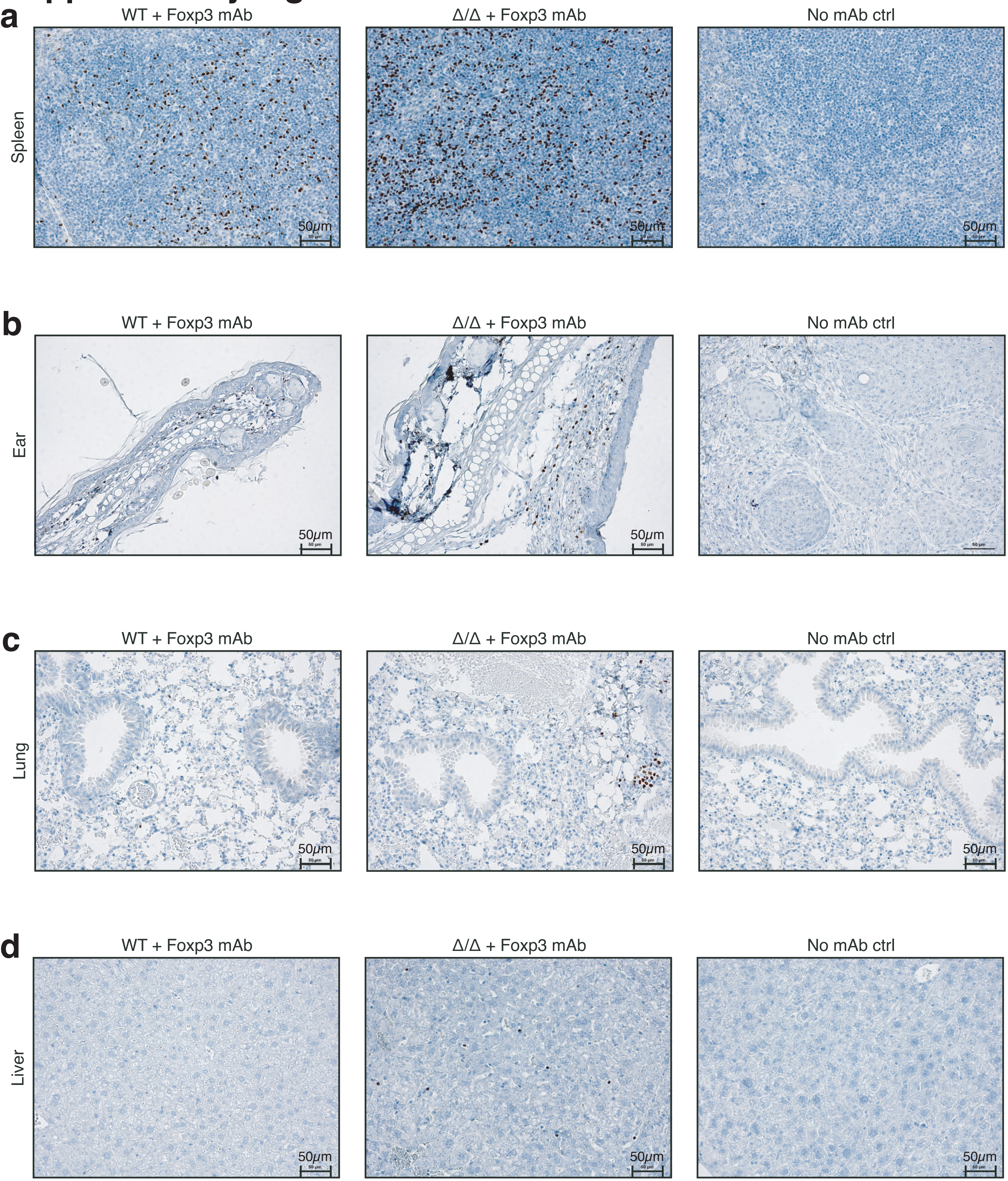

Supplementary Figure 6. Foxp3 antibody staining on paraffin-embedded tissue sections. This supplementary figure is an extension of **Figure 1e** and **Figure 5f**. Paraffin-embedded tissue sections were stained with a Foxp3 antibody, combined with Hematoxylin staining. (**a**) Staining of spleen tissue from wildtype animal (left) and affected Δ/Δ animal (middle) with Foxp3 antibody, same sample as in **Figure 1e**; control staining without Foxp3 antibody to the right. (**b**) Foxp3 staining ear tissue from wildtype animal (left) and affected Δ/Δ animal (middle) with Foxp3 antibody; control skin staining without Foxp3 antibody to the right. (**c**) Foxp3 staining of lung tissue from wildtype animal (left) and affected Δ/Δ animal (middle) with Foxp3 antibody as in **Figure 5f**; control staining without Foxp3 antibody to the right. (**d**) Foxp3 staining of liver tissue from wildtype animal (left) and affected Δ/Δ animal (middle) with Foxp3 antibody; control staining without Foxp3 antibody to the right.

Supplementary Figure 7

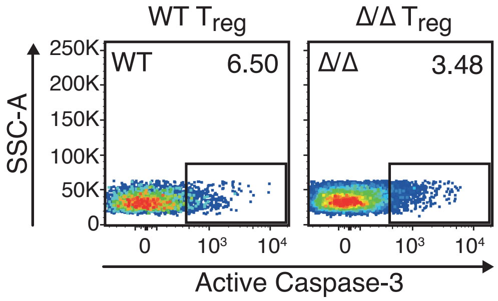

Supplementary Figure 7. Analysis of active-caspase 3 in T_{reg} cells from affected Δ/Δ animals vs WT animals. Representative dot plots illustrating expression of active caspase-3 in T_{reg} cells ($CD3^{+}CD4^{+}CD8^{-}CD25^{+}Foxp3^{+}$). Source data are provided as a Source Data file.

Supplementary Figure 8

a

Motifs found in 3,392 peaks up in Δ/Δ

Motif	p-value	Family	Score
CAGATAAGAATA	10^{-68}	Gata3<br>Gata4<br>Gata6	0.95<br>0.95<br>0.94
AAATGGGTCA	10^{-46}	Ap1<br>CoupTf<br>Fra2	0.76<br>0.76<br>0.74
ACTTCCTG	10^{-40}	Erg<br>Ets1<br>Elf3	0.95<br>0.94<br>0.93
ATGACCACATCA	10^{-35}	Gata<br>Runx<br>Runx2	0.69<br>0.69<br>0.67
GGCCACACCC	10^{-32}	Eklf<br>Klf4<br>Klf5	0.93<br>0.90<br>0.88
ATCAGCAA	10^{-26}	MafF<br>MafA<br>Cux2	0.73<br>0.70<br>0.62

b

Motifs found in 10816 peaks up in WT

Motif	p-value	Family	Score
ACTTCCTC	10^{-116}	Pu1(Ets)<br>Ehf(Ets)<br>Etv2(Ets)	0.85<br>0.83<br>0.82
CCTTTGATGT	10^{-106}	Tcf3(Hmg)<br>Tcf4(Hmg)<br>Chr	0.95<br>0.95<br>0.71
TTTCTCAGAA	10^{-71}	Stat5(Stat)<br>Stat1(Stat)<br>Stat4(Stat)	0.97<br>0.90<br>0.87
TGACATTTAA	10^{-49}	Nur77(N)<br>Esrrb(N)<br>Erra(N)	0.82<br>0.72<br>0.69
CCCCCCCC	10^{-47}	Maz(Z)<br>Klf3(Z)<br>Znf467(Z)	0.89<br>0.75<br>0.74
GTCACGTG	10^{-39}	Bmal1(bH)<br>Clock(bH)<br>Usf2(bH)	0.80<br>0.80<br>0.79
CGTGAGAA	10^{-27}	Rbpj1<br>Tbet<br>Tbr1	0.84<br>0.75<br>0.70

c

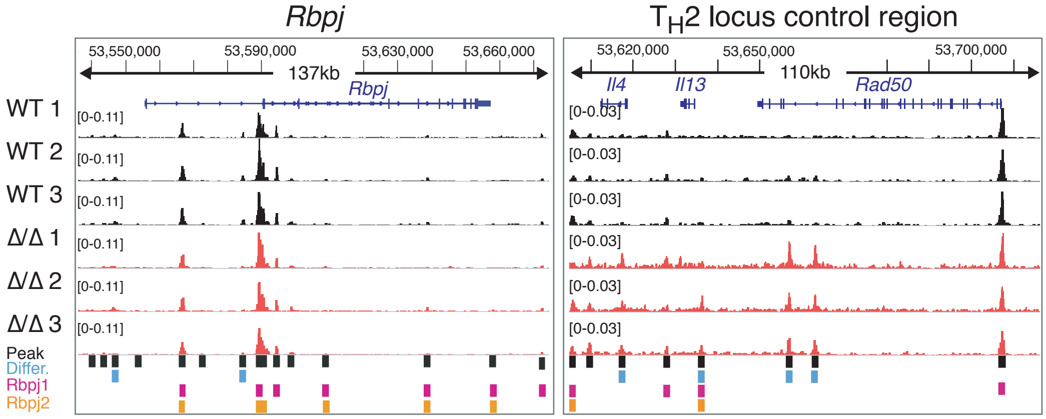

Supplementary Figure 8. Motif analysis and ATAC-seq. This supplementary figure is an extension of **Figure 8**. (a) *De novo* motif analysis in 3,392 peaks up in Δ/Δ T_{reg} cells. Top enriched *de novo* motifs are shown, along with the corresponding p-value and the three most-similar known motifs (similarity score from 0-1, with 1 indicating an exact match). (b) *De novo* motif analysis in 10,816 peaks up in WT T_{reg} cells. Top enriched *de novo* motifs are shown, along with the corresponding p-value and the three most-similar known motifs (similarity score from 0-1, with 1 indicating an exact match). (**c**) ATAC-seq genome browser tracks for two genes, *Rbpj* and the T_H2 locus control region, with WT Klrg1$^-$ T_{reg} cell data in black and Δ/Δ Klrg1$^+$ T_{reg} cell data in red. Gene information is shown on top, along with the genomic location. Height indicates normalized ATAC-seq signal, the scale shown in brackets. All samples are group-normalized to allow peak height comparison. Below, all peaks (black squares), differential peaks (blue squares), instances of the *de-novo* Rbpj binding motif (purple) or the literature-based Rbpj motif (orange) are shown. Source data are provided as a Source Data file.

Supplementary Table 1. List of antibodies, primers, critical chemicals and kits

Antibodies		
Pacific Blue anti-mouse CD3 antibody	Biolegend	AB_2028475
Brilliant Violet 711 anti-mouse CD3 antibody	Biolegend	AB_2563945
Purified anti-mouse CD3 antibody	Biolegend	AB_312667
APC anti-mouse CD4 antibody	Biolegend	AB_312719
APC/Cy7 anti-mouse CD4 antibody	Biolegend	AB_312699
Biotin anti-mouse CD4 antibody	Biolegend	AB_312711
Brilliant Violet 421 anti-mouse CD4 antibody	Biolegend	AB_11219790
FITC anti-mouse CD4 antibody	Biolegend	AB_312713
Brilliant Violet 711 anti-mouse CD4 antibody	Biolegend	AB_2562099
Brilliant Violet 605 anti-mouse CD4 antibody	Biolegend	AB_2563054
PE anti-mouse CD4 antibody	Biolegend	AB_312715
PE/Cy7 anti-mouse CD4 antibody	Biolegend	AB_312729
PerCP/Cy5.5 anti-mouse CD4 antibody	Biolegend	AB_893326
Brilliant UV 395 anti-mouse CD4 antibody	BD Biosciences	Cat# 563790
Brilliant UV 737 anti-mouse CD4 antibody	BD Biosciences	Cat# 564933
Biotin anti-mouse CD8a antibody	Biolegend	AB_312743
Brilliant Violet 605 anti-mouse CD8a antibody	Biolegend	AB_2562609
PE/Cy7 anti-mouse CD8a antibody	Biolegend	AB_312761
PerCP/Cy5.5 anti-mouse CD8a antibody	Biolegend	AB_2075238
Biotin anti-mouse/human CD11b antibody	Biolegend	AB_312787
Biotin anti-mouse CD11c antibody	Biolegend	AB_313773
APC/Cy7 anti-mouse CD19 antibody	Biolegend	AB_830707
Biotin anti-mouse CD19 antibody	Biolegend	AB_313639
APC anti-mouse CD25 antibody	Biolegend	AB_312861
Biotin anti-mouse CD25 antibody	Biolegend	AB_312853
PE anti-mouse CD25 antibody	Biolegend	AB_312857
PE/Cy7 anti-mouse CD25 antibody	Biolegend	AB_312865
Brilliant Violet 711 anti-mouse CD25 antibody	Biolegend	AB_2564130
Pacific Blue anti-mouse/human CD44 antibody	Biolegend	AB_493683
Brilliant Violet 421 anti-mouse/human CD44 ab	Biolegend	AB_10895752
Brilliant Violet 605 anti-mouse/human CD44 ab	Biolegend	AB_2562451
Brilliant Violet 421 anti-mouse CD45 antibody	Biolegend	AB_10899570
APC/Cy7 anti-mouse CD45 antibody	Biolegend	AB_312981
Pacific Blue anti-mouse CD45 antibody	Biolegend	AB_493535
APC anti-mouse CD62L antibody	Biolegend	AB_313099
APC/Cy7 anti-mouse CD62L antibody	Biolegend	AB_830799
PerCP/Cy5.5 anti-mouse CD62L antibody	Biolegend	AB_2285839
Alexa Fluor 647 anti-mouse CD103 antibody	Biolegend	AB_535952
PE anti-mouse CD103 antibody	Biolegend	AB_1133989
Brilliant Violet 605 anti-mouse CD127 (IL-7Rα) ab	Biolegend	AB_2562114
Brilliant Violet 421 anti-mouse CD127 (IL-7Rα) ab	Biolegend	AB_11218800
PE/Cy7 anti-mouse I-A/I-E antibody	Biolegend	AB_2290801
Pacific Blue anti-mouse I-A/I-E antibody	Biolegend	AB_493527
APC/Cy7 anti-mouse I-A/I-E antibody	Biolegend	AB_1659252
PE anti-mouse/human KLRG1 (MAFA) antibody	Biolegend	AB_10574005
Brilliant Violet 421 anti-mouse/human KLRG1 ab	Biolegend	AB_2565613
Brilliant Violet 605 anti-mouse/human KLRG1 ab	Biolegend	AB_2563357
Brilliant Violet 421 anti-mouse IL-33Ra (ST2)	Biolegend	AB_2565634
PE anti-mouse IL-33Ra (ST2)	Biolegend	AB_2561915
Anti-Mouse/Rat Foxp3 Alexa Fluor 647 antibody	Biolegend	AB_763538

Anti-Mouse/Rat Foxp3 Biotin antibody	Biolegend	AB_763540
Anti-Mouse/Rat Foxp3 PE antibody	Biolegend	AB_465936
Alexa Fluor 647 anti-Gata-3 antibody	Biolegend	AB_2563217
PE anti-Gata-3 antibody	Biolegend	AB_2562723
PE anti-mouse IL-2 antibody	Biolegend	AB_315302
PE anti-mouse IL-4 antibody	Biolegend	AB_315318
PE anti-mouse IL-5 antibody	Biolegend	AB_315327
PE anti-mouse IL-10 antibody	Biolegend	AB_315362
PE anti-mouse IL-13 antibody	eBiosciences	AB_763561
PE anti-mouse IL-17 antibody	Biolegend	AB_315463
PE anti-mouse IFNg antibody	Biolegend	AB_315402
Cxcr-5	Biolegend	AB_2562208
PD-1	Biolegend	AB_1877231
PE anti-mouse human Helios antibody	Biolegend	AB_10660749
PE anti-mouse CD85k (gp49) ILT3 antibody	Biolegend	AB_2561654
AF647 anti-mouse CD85k (gp49) ILT3 antibody	Biolegend	AB_2562044
PE/Cy7 anti-mouse CD273 (PD-L2) antibody	Biolegend	AB_2728123
BV510 anti-mouse CD11c antibody	Biolegend	AB_2562016
PE anti-mouse IRF4 antibody	Biolegend	AB_2563005
Goat Anti-Rabbit IgG (H+L) antibody, AF 647	Thermo Fisher	AB_10562581
Fixable Viability Dye eFluor 506	eBioscience	Cat# 65-0866-18
Mouse IgM control antibody	BD Biosciences	AB_393980
Mouse IgG1 control antibody	BD Biosciences	AB_479649
Mouse IgG2a control antibody	BD Biosciences	AB_571927
Mouse IgG2b control antibody	Santa Cruz Biotech	AB_626950
Mouse IgG3	Sigma Aldrich	Cat# 99043001
Mouse IgE	BD Biosciences	AB_479637
Goat anti-mouse IgM peroxidase	Southern Biotech	Cat# 1020-05
Goat anti-mouse IgG1 peroxidase	Southern Biotech	Cat# 1070-05
Goat anti-mouse IgG2a peroxidase	Southern Biotech	Cat# 1080-05
Goat anti-mouse IgG2b peroxidase	Southern Biotech	Cat# 1090-05
Goat anti-mouse IgG3 peroxidase	Southern Biotech	Cat# 1100-05
Donkey anti-mouse IgG antibody	Santa Cruz	AB_641171
APC/Cy7 Streptavidin	Biolegend	Cat# 405208
eFluro450 Streptavidin	eBioscience	Cat# 48-4317-82
FITC Streptavidin	Biolegend	Cat# 405201
PE Streptavidin	Biolegend	Cat# 405204
PE/Cy7 Streptavidin	Biolegend	Cat# 405206
PerCP/Cy5.5 Streptavidin	Biolegend	Cat# 405214
Brilliant UV 395 Streptavidin	BD Biosciences	Cat# 564176
Brilliant UV 737 Streptavidin	BD Biosciences	Cat# 564293
APC Streptavidin	Biolegend	Cat# 405207
Brilliant Violet 421 Streptavidin	Biolegend	Cat# 504421
Anti-rat HRP (pre-mixed)	Histofine	Cat# 41431 1F
**Primers & Probes**		
Taqman Probe for *Hprt*	Thermo Fisher	Mm01318746_g1
Taqman Probe for *Il2ra*	Thermo Fisher	Mm01340213_m1
Taqman Probe for *Foxp3*	Thermo Fisher	Mm00475162_m1
Taqman Probe for *Il7r*	Thermo Fisher	Mm00434295_m1
Taqman Probe for *Pparg*	Thermo Fisher	Mm01184322_m1
Taqman Probe for *Tbx21*	Thermo Fisher	Mm00450960_m1
Taqman Probe for *Irf4*	Thermo Fisher	Mm00516431_m1

Taqman Probe for *Gata3*	Thermo Fisher	Mm00484683_m1
Taqman Probe for *Il10*	Thermo Fisher	Mm01288386_m1
Taqman Probe for *Il1rl1*	Thermo Fisher	Mm00516117_m1
Taqman Probe for *Rora*	Thermo Fisher	Mm01173766_m1
Taqman Probe for *Rbpj* Exon 1-2	Thermo Fisher	Mm00770450_m1
Taqman Probe for *Rbpj* Exon 6-7	Thermo Fisher	Mm01217627_g1
Taqman Probe for *Dtx1* Exon 9-10	Thermo Fisher	Mm00492297_m1
Bisulfite-DNA primer for *Foxp3* R3 (TSDR)	Reverse Primer	TGGGTTTTTTTGGTATTTAAGAAAG
Bisulfite-DNA primer for *Foxp3* R3 (TSDR)	Forward Primer	AAAAAACAAATAATCTACCCCACAA
Rbpj Exon 1-2 Sybr Primer	Forward Primer	ATGCCCTCCGGTTTTCCTC
Rbpj Exon 1-2 Sybr Primer	Reverse Primer	GGACAAGCCCTCCGAGTAGT
Rbpj Exon 6-7 Sybr Primer	Forward Primer	TTTCCACGCCAGTTCACAACA
Rbpj Exon 6-7 Sybr Primer	Reverse Primer	TCTGCCCGTAATGGATGTAGC
**Critical Chemicals**		
DNAse	Roche	Cat# 11284932001
5mC-dNTP mix	Zymo Research	Cat# D1030
Power SYBR Green Master Mix	Thermo Fisher	Cat# 4367659
Taqman Gene Expression Master Mix	Thermo Fisher	Cat# 4359016
SuperScript II Reverse Transcriptase	Thermo Fisher	Cat# 18064071
Oligo d(T) 12-18 Primer	Thermo Fisher	Cat# 18418012
Anti-biotin Microbeads	Miltenyi Biotec	Cat# 130-090-385
Dynabeads Mouse T-Activator CD3/CD28	Thermo Fisher	Cat# 11456D
Mouse T-cell activation and expansion kit	Miltenyi Biotec	Cat# 130-093-627
Purified anti-mouse IFN-gamma antibody	Biolegend	AB_315396
Recombinant murine IL-2	Peprotech	Cat# 212-12
Recombinant murine IL-4	Peprotech	Cat# 214-14
Recombinant murine IL-7	Peprotech	Cat# E1915
Recombinant murine IL-33	Biolegend	Cat# 280504
Lipopolysaccharide (E.*coli* 0127:B8)	Sigma	Cat# L3129
PMA/Ionomycin stim cocktail plus transport inh.	eBioscience	Cat# 00-4975-03
Transport inhibitor control	eBioscience	Cat# 00-4980-03
BD Fixation Buffer	BD Biosciences	Cat# 554655
BD PermBuffer III	BD Biosciences	Cat# 558050
Peroxidase-blocking reagent	Dako	Cat# S2023
Antibody diluent	Dako	Cat# S2022
DAB+	Dako	Cat# K5007
Wash Buffer	Dako	Cat# 3006
Hematoxylin	Merck	Cat# 1051750500
**Critical Commercial Kits**		
RNEasy Mini Kit	Quiagen	Cat# 74104
RNEasy Plus Micro Kit	Quiagen	Cat# 74034
DNEasy Blood and Tissue kit	Quiagen	Cat# 69504
EpiTect Bisulfite conversion kit	Zymo Research	Cat# 59104
NEBNext High Fidelity Mix	NEB	M0541
BCA Protein Assay Kit	Thermo Fisher	Cat# 23227
Chromogenic Detection kit CN/DAB	Thermo Fisher	Cat# 34000
PureLink Quick Gel Extraction Kit	Thermo Fisher	Cat# K210012
Foxp3 / Transcription Factor Staining Buffer Set	eBioscience	Cat# 00-5523-00
Active Caspase-3 kit	Abcam	Cat# 65617
Mouse IgE ELISA kit	BD Biosciences	Cat# 555248

Illumina Nextera DNA library prep kit	Illumina	Cat# 15028212
