# Supplementary material for: Rbpj expression in regulatory T cells is critical for restraining TH2 responses
Source: Nat Commun. 2019 Apr 8;10:1621. doi: 10.1038/s41467-019-09276-w (PMC6453958; doi:10.1038/s41467-019-09276-w)
Supplement: Supplementary file 2 — Reporting Summary [file 41467_2019_9276_MOESM2_ESM.pdf]

nature research

Corresponding author(s): Prof. Dr. Markus Feuerer

Reporting Summary

Nature Research wishes to improve the reproducibility of the work that we publish. This form provides structure for consistency and transparency in reporting. For further information on Nature Research policies, see Authors & Referees and the Editorial Policy Checklist.

Statistical parameters

When statistical analyses are reported, confirm that the following items are present in the relevant location (e.g. figure legend, table legend, main text, or Methods section).

n/a	Confirmed	
☐	☒	The exact sample size (*n*) for each experimental group/condition, given as a discrete number and unit of measurement
☐	☒	An indication of whether measurements were taken from distinct samples or whether the same sample was measured repeatedly
☐	☒	The statistical test(s) used AND whether they are one- or two-sided<br>*Only common tests should be described solely by name; describe more complex techniques in the Methods section.*
☒	☐	A description of all covariates tested
☐	☒	A description of any assumptions or corrections, such as tests of normality and adjustment for multiple comparisons
☒	☐	A full description of the statistics including central tendency (e.g. means) or other basic estimates (e.g. regression coefficient) AND variation (e.g. standard deviation) or associated estimates of uncertainty (e.g. confidence intervals)
☒	☐	For null hypothesis testing, the test statistic (e.g. *F*, *t*, *r*) with confidence intervals, effect sizes, degrees of freedom and *P* value noted<br>*Give P values as exact values whenever suitable.*
☒	☐	For Bayesian analysis, information on the choice of priors and Markov chain Monte Carlo settings
☒	☐	For hierarchical and complex designs, identification of the appropriate level for tests and full reporting of outcomes
☒	☐	Estimates of effect sizes (e.g. Cohen's *d*, Pearson's *r*), indicating how they were calculated
☐	☒	Clearly defined error bars<br>*State explicitly what error bars represent (e.g. SD, SE, CI)*

Our web collection on statistics for biologists may be useful.

Software and code

Policy information about availability of computer code

Data collection	All code used to collect data has already been published and is properly referenced in the materials and methods section.
Data analysis	All code used to analyze our data has already been published and is properly referenced in the materials and methods section.

For manuscripts utilizing custom algorithms or software that are central to the research but not yet described in published literature, software must be made available to editors/reviewers upon request. We strongly encourage code deposition in a community repository (e.g. GitHub). See the Nature Research guidelines for submitting code & software for further information.

Data

Policy information about availability of data

All manuscripts must include a data availability statement. This statement should provide the following information, where applicable:

- Accession codes, unique identifiers, or web links for publicly available datasets
- A list of figures that have associated raw data
- A description of any restrictions on data availability

Gene microarray data, RNA sequencing and ATAC sequencing data are deposited at the Gene Expression Omnibus with the Accession Number GSE119169 . A statement about the accession codes has been added to the materials and methods section. The data have been released as of 31st January 2019.

Field-specific reporting

Please select the best fit for your research. If you are not sure, read the appropriate sections before making your selection.

☒ Life sciences ☐ Behavioural & social sciences

For a reference copy of the document with all sections, see nature.com/authors/policies/ReportingSummary-flat.pdf

Life sciences

Study design

All studies must disclose on these points even when the disclosure is negative.

Sample size	For in-vitro experiment, we used a small sample size of 3-5 animals per replicate.
Data exclusions	No data were excluded from the analysis.
Replication	We repeated experiments, where applicable. Complex mouse experiments, such as infection with S. ratti, were performed in two cohorts. Complex sequencing experiments were validated with a second method, e.g. validation of RNA-sequencing by protein or qPCR measurement.
Randomization	No randomization.
Blinding	No blinding.

Materials & experimental systems

Policy information about availability of materials

n/a	Involved in the study
☒	☐ Unique materials
☐	☒ Antibodies
☒	☐ Eukaryotic cell lines
☐	☒ Research animals
☒	☐ Human research participants

Antibodies

Antibodies used	We supply an extensive antibody list in the supplemental table. This list contains supplier information as well as antibody registry number.
Validation	Antibodies have been used as recommended by manufacturer.

Research animals

Policy information about studies involving animals; ARRIVE guidelines recommended for reporting animal research

Animals/animal-derived materials	Wildtype C57BL/6, congenic B6.SJL-PtprcaPepcb/BoyCrl (CD45.1+), and congenic B6.PL-Thy1a/CyJ (CD90.1+) mice were obtained from Charles River Breeding Laboratories (Wilmington, MA, USA) or the Jackson Laboratory (Bar Harbor, ME, USA). B6N.129(Cg)-Foxp3tm3Ayr mice (Foxp3.IRES-DTR/GFP)48 were bred to CD45.1+ or CD90.1+ mice in the animal facility of the German Cancer Research Center (DKFZ). B6.129(Cg)-Foxp3tm4(YFP/cre)Ayr/J, Jackson (FOXP3.IRES-YFP/Cre) were crossed to Rbpjfl/fl mice to specifically delete Rbpj in Treg cells. Age matched littermate controls (Foxp3Cre,YFP-positive and wildtype for the Rbpj alleles) were used throughout the study. Details about hygiene status and barrier breeding conditions in the following paragraph. Rag2-deficient (B6-Rag2tm1Fwa) lines were used to isolate protein for autoantibody detection. All animals were housed under specific pathogen-free conditions at the respective animal care facilities, and the governmental committee for animal experimentation approved all experiments involving animals.

Method-specific reporting

n/a	Involved in the study
☒	☐ ChIP-seq
☐	☒ Flow cytometry
☒	☐ Magnetic resonance imaging

Flow Cytometry

Plots

Confirm that:

☐ The axis labels state the marker and fluorochrome used (e.g. CD4-FITC).

☒ The axis scales are clearly visible. Include numbers along axes only for bottom left plot of group (a 'group' is an analysis of identical markers).

☒ All plots are contour plots with outliers or pseudocolor plots.

☒ A numerical value for number of cells or percentage (with statistics) is provided.

Methodology

Sample preparation	Secondary lymphoid tissues (lymph nodes, spleen) and peripheral tissues were harvested and processed as described in M&M.
Instrument	Samples were measured on BD LSR II or BD FACS ARIA II instruments.
Software	Data were analyzed with FlowJo version 10.
Cell population abundance	We obtained post-sort controls of relevant populations. Sequencing was only conducted upon high-efficiency and high-purity sorting of target cell populations. If applicable, target cells were pre-enriched prior to sorting.
Gating strategy	We used commonly-accepted gating strategies to identify lymphocytes (FSC vs SSC), single cells (FSC-H vs FSC-W), and viable cells (dead cell-dye negative). Gating for common strong surface proteins such as CD3, CD4, CD8, or CD44 according to accepted standards. More complex gating, e.g. CD25 and Foxp3-GFP for Treg cells or ST-2 and Klrg-1 for tisTregST2 cells is shown in the main figures.

☐ Tick this box to confirm that a figure exemplifying the gating strategy is provided in the Supplementary Information.
